# Impact of the HIV-1 genetic background and HIV-1 population size on the evolution of raltegravir resistance

**DOI:** 10.1186/s12977-017-0384-z

**Published:** 2018-01-05

**Authors:** Axel Fun, Thomas Leitner, Linos Vandekerckhove, Martin Däumer, Alexander Thielen, Bernd Buchholz, Andy I. M. Hoepelman, Elizabeth H. Gisolf, Pauline J. Schipper, Annemarie M. J. Wensing, Monique Nijhuis

**Affiliations:** 1 Department of Medical Microbiology, Virology, University Medical Center Utrecht, Heidelberglaan 100, HP G04.614, 3584 CX Utrecht, The Netherlands; 2 Theoretical Biology and Biophysics, Los Alamos National Laboratory, Los Alamos, NM USA; 3 Department of General Internal Medicine and Infectious Diseases, Ghent University Hospital, Ghent, Belgium; 4 Institute of Immunology and Genetics, Kaiserslautern, Germany; 5 Max Planck Institute for Informatics, Saarbrücken, Germany; 6 Pediatric Clinic, University Medical Center Mannheim, Mannheim, Germany; 7 Department of Internal Medicine and Infectious Diseases, University Medical Center Utrecht, Utrecht, The Netherlands; 8 Department of Internal Medicine, Rijnstate Hospital, Arnhem, The Netherlands

## Abstract

**Background:**

Emergence of resistance against integrase inhibitor raltegravir in human immunodeficiency virus type 1 (HIV-1) patients is generally associated with selection of one of three signature mutations: Y143C/R, Q148K/H/R or N155H, representing three distinct resistance pathways. The mechanisms that drive selection of a specific pathway are still poorly understood. We investigated the impact of the HIV-1 genetic background and population dynamics on the emergence of raltegravir resistance. Using deep sequencing we analyzed the integrase coding sequence (CDS) in longitudinal samples from five patients who initiated raltegravir plus optimized background therapy at viral loads > 5000 copies/ml. To investigate the role of the HIV-1 genetic background we created recombinant viruses containing the viral integrase coding region from pre-raltegravir samples from two patients in whom raltegravir resistance developed through different pathways. The in vitro selections performed with these recombinant viruses were designed to mimic natural population bottlenecks.

**Results:**

Deep sequencing analysis of the viral integrase CDS revealed that the virological response to raltegravir containing therapy inversely correlated with the relative amount of unique sequence variants that emerged suggesting diversifying selection during drug pressure. In 4/5 patients multiple signature mutations representing different resistance pathways were observed. Interestingly, the resistant population can consist of a single resistant variant that completely dominates the population but also of multiple variants from different resistance pathways that coexist in the viral population. We also found evidence for increased diversification after stronger bottlenecks. In vitro selections with low viral titers, mimicking population bottlenecks, revealed that both recombinant viruses and HXB2 reference virus were able to select mutations from different resistance pathways, although typically only one resistance pathway emerged in each individual culture.

**Conclusions:**

The generation of a specific raltegravir resistant variant is not predisposed in the genetic background of the viral integrase CDS. Typically, in the early phases of therapy failure the sequence space is explored and multiple resistance pathways emerge and then compete for dominance which frequently results in a switch of the dominant population over time towards the fittest variant or even multiple variants of similar fitness that can coexist in the viral population.

## Background

Currently, viral replication is successfully suppressed in the majority of HIV-infected patients treated with combination antiretroviral therapy (cART) [1]. However, virological failure associated with the emergence of drug resistant viruses may still limit the success of cART. The emergence of drug resistance in HIV is a direct consequence of the high error-rate of the HIV reverse transcriptase (RT) enzyme [2–4]. The frequent incorrect nucleotide incorporations result in evolution of the viral population and generate a myriad of viral variants upon which selective forces may act. The population size and replication rate are important viral parameters that contribute to the probability that resistance emerges and to how HIV-1 drug resistance evolves [5–9].

Integrase inhibitors comprise a class of antiretroviral drugs that specifically prevent the integration of the viral genome into the human genome. Raltegravir is the first representative of a class of integrase inhibitors that target the strand transfer reaction (INSTIs) of the viral DNA into the host genome which is performed by the viral enzyme integrase. Like other INSTIs, raltegravir preferentially binds and inhibits the viral DNA-integrase complex (intasome) over unbound integrase [10–12].

It was the first integrase inhibitor used in clinical practice (since 2007) but was recently registered by the FDA for once daily dosing [13] and is very well tolerated [14] due to a low toxicity profile [15]. Resistance is commonly associated with selection of one of the signature raltegravir resistance mutations Y143C/R/H, Q148H/K/R or N155H [16–18]. Mutations at each of these three amino acids represent a distinct resistance pathway and all signature mutations are associated with reductions in viral replication [17, 19, 20]. Accumulation of secondary resistance mutations is often associated with a greater loss of drug susceptibility and/or improved viral fitness [21–23]. Different mutational combinations vary greatly in their impact on raltegravir susceptibility and viral replication. In general, substitutions at amino acid position 148 confer higher levels of resistance than substitutions at amino acid Y143 or N155. The G140S plus Q148H combination is considered the most resistant variant and has little effect on viral replication. The resistance patterns observed in HIV-1 patients on a raltegravir containing regimen are very diverse and Q148H/K/R (usually with G140A/C/S and/or E138A/K) and N155H (often together with E92Q or V151I) mutations are observed more frequently than Y143 mutations [24]. The different resistance pathways are believed to be mutually exclusive and multiple primary mutations (especially 148 + 155 mutations) are generally not observed on the same viral genome [25]. Remarkably, replacement of the dominant resistant population by a viral population with a completely different resistance pattern during continuous non-suppressive INSTI therapy has been observed [26–29].

The mechanisms that drive selection and switching of resistance pathways are inadequately understood. Understanding these mechanisms is essential in view of other INSTIs that are in clinical use (elvitegravir and dolutegravir) or in clinical trial (bictegravir and cabotegravir), since their resistance profiles partially overlap with that of raltegravir [25]. For instance, raltegravir resistance mutations Q148H/K/R and N155H show a high level of cross-resistance with elvitegravir, but elvitegravir susceptibility is unaffected by Y143 mutations [30, 31], a difference that is beautifully explained by crystal structures of the intasome in presence of raltegravir or elvitegravir [11]. The VIKING studies demonstrated dolutegravir’s superior resistance profile attested by sustained activity against all raltegravir resistant variants, except for viruses with a mutation at amino acid 148 in combination with at least one secondary mutation at position 138 and/or 140 [32–34]. These observations were corroborated by in vitro analysis of resistance profiles [35–37] which also uncovered two atypical INSTI resistance mutations (G118R and F121Y) that confer pan-INSTI resistance [38, 39].

We investigated the impact of the HIV-1 genetic background and population size on the evolution of raltegravir resistance and their role in determining selection of a particular resistance pathway. Using next-generation sequencing (NGS) we analyzed patient-derived viral integrase sequences from samples taken before and during raltegravir therapy failure. Frequency and dynamics analysis of the deep sequencing data was used to evaluate intra-patient evolution of resistance.

To investigate the role of the genetic background we generated recombinant viruses containing the viral integrase CDS from pre-raltegravir samples from two patients experiencing virological failure receiving raltegravir therapy. With these recombinant viruses we conducted multiple low multiplicity of infection (MOI) in vitro selections in parallel, with the advantage that all resistant variants generated are allowed to replicate and different raltegravir resistance pathways can be identified.

## Results

### During raltegravir resistance development, multiple resistant variants emerge that compete to become the dominant variant

We studied five patients who initiated raltegravir therapy as part of a cART regimen and subsequently demonstrated virological failure to the raltegravir containing regimen. Of note, all 5 patients were heavily pre-treated and raltegravir was part of a (partly) compromised backbone. This may partially explain the moderate virological responses and limited therapy success in these patients. Patient (Pt) 1, Pt2, Pt3 and Pt5 were infected with HIV-1 subtype B strains, Pt4 was infected with a HIV-1 CRF02_AG strain. All patients started raltegravir therapy with viral loads > 5000 copies/ml (c/ml) of HIV-1 RNA, as measured in the last viral load test before initiation of raltegravir therapy. To investigate if the emerged resistant variants existed as minority variants before raltegravir therapy and how the resistant population evolved we analyzed longitudinal samples from these patients by NGS.

#### Patient 1

**Fig. 1:**
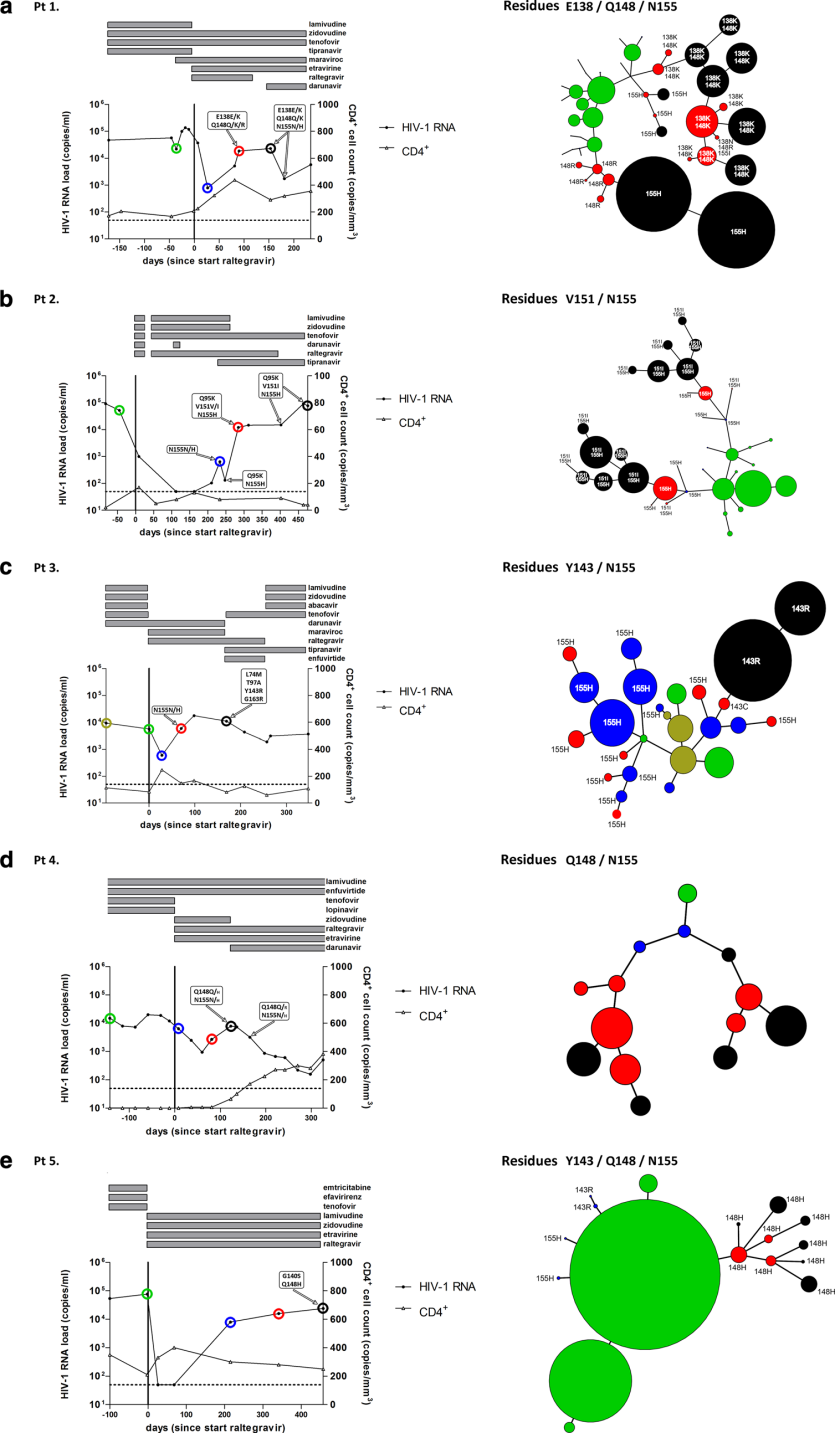
Development of raltegravir resistance during raltegravir containing cART. Left hand panels: therapy history, HIV-1 RNA load, CD4^+^ cell count and resistance mutations detected by population sequencing of five patients receiving raltegravir therapy. All viral load measurements are marked by a solid black circle. The CD4^+^ cell counts are represented by open triangles. Samples analyzed by 454 deep sequencing are marked by colored circles. Resistance mutations detected by Sanger population sequencing are indicated in boxes. Only raltegravir resistance associated mutations are given. Right hand panels: evolution of resistance pathways, deep sequence analysis of the integrase core domain. Data was obtained by 454 pyrosequencing. Relevant resistance mutations are indicated at the respective nodes. Figures were generated using the nucleotide sequences and the redundancy-level for calling a variant was set at 80. No mutation information indicates wild-type amino acids. The size of each node is scaled to reflect the relative abundance and viral load at each time point and patient. Time points are indicated by color and correspond to the colored circles in the left hand panels: green is the baseline sample, blue the first sample after raltegravir therapy initiation; red is the second sample after raltegravir therapy initiation; black is the final sample after raltegravir therapy initiation. In patient 3 gold is another baseline sample predating the green sample

**Fig. 2:**
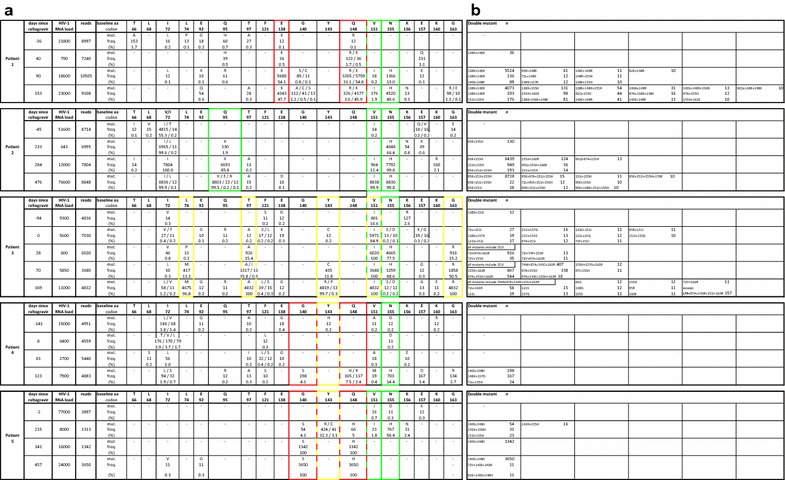
Analysis of the longitudinal 454 deep sequencing data. **a** Analysis of the frequency of all non-synonymous mutations detected by deep sequencing at the 18 codons associated with raltegravir resistance. Only unique variants with a minimum of 10 reads were included in the analysis. Total number of reads and the proportion of reads containing the denoted mutations relative to the total number of reads are given. Mutations of interest are highlighted by colored boxes. Similar colored boxes are mutations that appeared to be on the same genome. Red boxes indicate mutations from the Q148 pathway, yellow boxes indicate the Y143 pathway and green boxes the N155 pathway. **b** Sequences containing multiple mutations are shown. Double mutants are sorted by frequency

HIV-1 RNA initially decreased after start of raltegravir containing cART (2.2 log decrease in HIV-1 RNA), but the viral load rebounded quickly after therapy initiation (< 81 days, Fig. 1a). Population sequencing revealed presence of raltegravir resistance mutations (E138E/K + Q148Q/K/R + N155H/H) and raltegravir was discontinued from the regimen after 124 days. NGS revealed very small populations at baseline containing Q148R and E138K (0.1% of the population, Fig. 2a), but they were not present on the same genomes (Fig. 2b). 40 days after start of raltegravir, virus with Q148R had increased to 1.7% of the population and virus with E138K + Q148K had increased to 0.5% (Fig. 2a, b). These two variants dominated the population after 90 days (red nodes, Fig. 1a) but a third resistant variant appeared, N155H which already comprised 13% of the population. In the subsequent sample (153 days after start of raltegravir, black nodes Fig. 1a) the N155H variant replaced the Q148R variant and dominated the population together with the E138K + Q148K variant. Surprisingly, 4 weeks after raltegravir discontinuation, wild-type virus had not reseeded the viral population which was consisted entirely of raltegravir resistant variants (Fig. 2).

#### Patient 2

Pt2 was off therapy for a few weeks due to toxicity related issues but a new five-drug regimen including raltegravir and darunavir resulted in a rapid decline of the viral load but was discontinued again because of darunavir-related toxicity (Fig. 1b). Shortly thereafter, the same regimen was restarted without darunavir resulting in a further decline of the viral load to undetectable levels. After brief virological suppression (HIV-1 RNA < 50 c/ml, a 3.0 log decrease in HIV-1 RNA), viral load rebounded and population sequencing showed gradual accumulation of raltegravir resistance mutations: initially primary resistance mutation N155H appeared followed by two secondary resistance mutations, first Q95K and later V151I. NGS revealed no major raltegravir resistance mutations at baseline or mutations at positions 143 or 148 at any time-point, not even as minority variants (Fig. 2).

#### Patient 3

Pt3 only showed a partial virological response to the raltegravir containing therapy (a 1.0 log decrease in HIV-1 RNA was observed) and full suppression was never achieved. The viral load rebounded shortly after start of raltegravir containing cART (< 70 days, Fig. 1c) and the viral population contained N155H mutants. In a later time-point, 169 days after start of raltegravir, this population was replaced by a variant with primary mutation Y143R and several secondary mutations including L74M, T97A and G163E. NGS revealed only secondary mutations in the sample before raltegravir therapy, mostly as very low frequency variants (Fig. 2), including T97A and Y143C (both 0.2%). In a sample taken 28 days later, 77.5% of the population contained N155H, 15.4% had T97A and variants with Y143C were not detected anymore. In the subsequent sample 70 days after start of raltegravir therapy, N155H had increased to 88.6% and Y143C had reappeared in 11.8% of the reads. Y143C and N155H did not appear to be on the same genome (Fig. 2b). After 169 days the resistant population had shifted dramatically, with Y143R making up 99.7% of the population and complete absence of N155H. Mutations L74M, T97A and G163R appeared in nearly the entire viral population.

#### Patient 4

Pt4 was also a partial responder (1.1 log decrease in HIV-1 RNA) in whom virological suppression was not achieved during 48 weeks of raltegravir containing therapy. Sanger sequencing revealed only very small (< 20%) populations of Q148H/Q148R and N155H and resistant variants never dominated the population (Fig. 1d). Before raltegravir therapy, mutation Y143H was detected at low frequency (0.2%) by NGS but did not appear in any of the later samples during raltegravir treatment. In samples 8 and 81 days after start of raltegravir therapy also no significant raltegravir resistance mutations were observed (Fig. 2a). However, in the sample 123 days after raltegravir therapy initiation several primary and secondary raltegravir resistance mutations occurred. N155H was found in 14.4%, of the population, Q148H in 7.5% of which approximately half (4.1%) in combination with G140S and the remainder in combination with E157D (Fig. 2b). Q148R was present in 2.4% of the population and G163R in 2.7%.

#### Patient 5

In addition to a compromised backbone, this patient also had sub-therapeutic levels of raltegravir levels due to a drug–drug interaction with rifampicin (an interaction unknown at time of raltegravir prescription). Despite the suboptimal levels of raltegravir, switching to a raltegravir containing regimen quickly resulted in complete viral suppression (< 26 days, 3.3 log decrease in HIV-1 RNA) but viral load rebounded within 21 weeks (Fig. 1e). Population sequencing (sample 457 days after initiation of raltegravir) demonstrated that virus with integrase substitutions G140S + Q148H completely dominated the viral population.

No major resistance mutations were detected at baseline by NGS. Interestingly, in the first sample during therapy failure (blue nodes Fig. 1e) mutations from all three major resistance pathways including double mutants with secondary mutations were observed with G140S + Q148H being the most frequently occurring double mutant (Fig. 2). In this sample the variant with mutation N155H was the dominant variant but was outcompeted in the subsequent sample (red nodes Fig. 1e) by double mutant G140S + Q148H.

### Evidence for elevated diversification following extinction from drug pressure on large viral populations

**Fig. 3:**
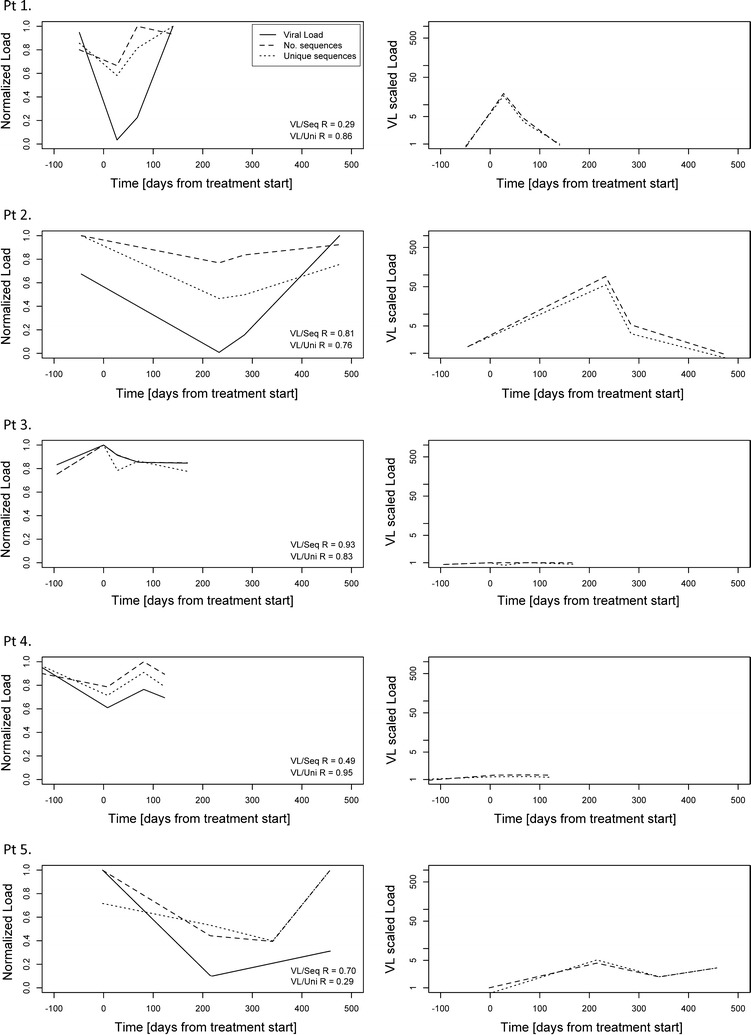
Viral load versus total number of sequences and number of unique variants. Left panels: the normalized (range 0–1) viral load, number of detected sequences and unique number of variants. The correlation coefficient, R, is indicated for the comparisons to the viral load. Right panels: the relative number of derived sequences and the number of unique variants relative to the viral load. Figures were generated using the nucleotide sequences and the redundancy-level for calling a variant was set at 80

To get an impression of the evolution of the viral population during raltegravir pressure we analyzed how the number of derived sequences and unique variants related to the viral load. To allow for easy comparisons, each of these measures were normalized on a 0–1 scale and plotted in the same graph (Fig. 3, left panels). In all patients, the total number of derived sequences and the number of unique variants detected correlated with the viral load (R = 0.29–0.95). However, in a few cases either the number of sequences detected (Pt1 and Pt4) or the number of unique variants (Pt5) showed weaker correlations and thus we also investigated the population diversity relative to the viral load. Therefore, the normalized number of sequences and unique variants were divided by the normalized viral load respectively (Fig. 3, right panels). Interestingly, in patients 1, 2 and 5, the proportion of unique sequence variants appeared to increase when the viral load dropped. This suggests a sudden diversifying pressure on the viral population. In contrast, in patients 3 and 4 there appeared to be no correlation between the relative number of unique variants and the viral load which coincided with the smallest reductions in viral load during raltegravir containing cART (Fig. 1c, d). These data indicate that the stronger the bottleneck is (i.e. largest reduction in viral load), the larger the effect is on subsequent diversification. Thus, extinction due to antiretroviral treatment appears to induce diversification.

### The genetic background is not paramount for the emerging INSTI resistance pathway

To investigate the role of the viral genetic background in determining the raltegravir resistance pathway we cloned the integrase coding region from pre-raltegravir therapy samples from two patients (Pt1 and Pt2) in an HXB2 reference background. These recombinant viruses were derived from amplicons of pre-raltegravir samples with a viral load of 21,800 and 51,600 c/ml respectively, creating libraries containing thousands of patient-derived sequences for both samples.

In these patients, raltegravir resistance developed through different pathways during raltegravir therapy. In Pt1, resistance developed initially through E138K + Q148K and later a second variant emerged with N155H (Fig. 1a). In Pt2, raltegravir resistance developed initially through N155H and was complemented by two secondary mutations, Q95K and V151I, which made up nearly 100% of the viral population during prolonged therapy failure (Fig. 1b). With the recombinant viruses of these raltegravir baseline samples and HXB2 reference virus (molecular clone from pHXB2AF, therefore a single sequence input per replicate) multiple in vitro selections were performed in parallel.

**Fig. 4:**
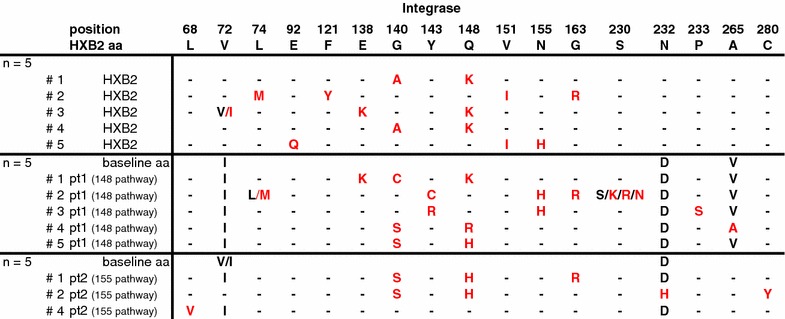
Raltegravir in vitro selections with patient-derived integrase recombinant viruses. Raltegravir concentration was doubled in each serial passage to a final concentration of 1024 nM of raltegravir in passage 10. All differences from the HXB2 reference sequence are given and mutations emerging during the raltegravir in vitro selections in the viral integrase coding region are indicated in red. The indicated mutations were detected by population sequencing of viral RNA in the culture supernatants from passage 10. Cultures 3 and 5 from Pt2 were discontinued due to failed virus propagation

All cultures were maintained for 10 serial passages to a final concentration of 1024 nM raltegravir. HXB2 virus predominantly selected mutation Q148K (in three out of the five independent cultures), N155H was selected once and in one culture only secondary resistance mutations emerged (Fig. 4). Two of the Q148K mutations were accompanied by G140A, the third by E138K. G140S was probably not selected because it required two nucleotide changes in this HXB2 background; G140A and E138K required just one. The N155H-virus additionally acquired mutations V151I and later E92Q. Amino acid substitutions at residue 143 were not observed. In vitro selections with Pt1 recombinant virus yielded amino acid substitutions at all three major resistance positions. Again, Q148 mutations dominated (3/5 cultures). Interestingly, in the two other cultures, N155H emerged in combination with amino acid substitutions at position 143 (one with Y143C and one with Y143R, Fig. 4). The mutations selected in vitro differed remarkably from what was observed in vivo. The difference between in vivo and in vitro resistance was even more profound for Pt2. In vivo, only mutations relating to the N155H pathway were observed and no other significant mutations were detected by deep sequencing. In contrast, in vitro only mutations from the Q148 pathway were detected (Fig. 4). Cultures #1 and #2 both developed raltegravir resistance through G140S + Q148H. Culture #1 initially selected G140S + Q148R but later switched to Q148H (not shown). Two of the five cultures were not able to replicate at higher raltegravir concentrations. When an earlier passage of both cultures was sequenced, no mutations in the viral integrase were found so these cultures were discontinued. A third culture only selected an L68V substitution, but this virus did not demonstrate phenotypic resistance when tested (data not shown).

## Discussion

We investigated the impact of the genetic background and viral population size on the development and evolution of raltegravir resistance in vitro and in vivo. Deep sequencing revealed presence of major raltegravir resistance mutations at baseline in 3/5 patients (patients (1, 3 and 4, Fig. 2a) at very low frequencies (≤ 0.2%). Only the major resistance mutation detected before therapy in Pt3, Y143C (0.2% in 2nd sample on day 0), ultimately became the dominant variant in the resistant population although it surprisingly disappeared from the subsequent sample to reappear in the 4th sample (11.8% on day 70). In the final sample (169 days) Y143C was not detected anymore but virus with mutation Y143R completely dominated the viral population. In the majority of subtype B viruses, the Y143R substitution requires two nucleotide changes (Y143 = TAC → TGC = 143C → CGC = 143R). So it appears that the Y143C variant detected at baseline facilitated selection of the more resistant Y143R variant [27] in this patient. In Pt 1, secondary resistance mutation E138K was detected at baseline (0.1%) and appeared to have acquired Q148K as in the subsequent sample a double mutant with E138K + Q148K was detected (0.5%) which persevered in the population (44.7% in the final sample). This suggests that minor variants present at baseline can play a role in the development and evolution of raltegravir resistance but are not essential for the emergence of resistance [40–42], which is also seen for other drug classes [43, 44].

Remarkably, in Pt1 a variant with major mutation N155H and no apparent secondary resistance mutations emerged after and then co-existed alongside the E138K + Q148K double mutant, each comprising roughly 50% of the viral population. This suggests a fitness advantage for the N155H single mutant over the E138K + Q148K double mutant. This is unexpected considering the in vitro observations regarding resistance and replication of these mutants [17, 45, 46]. A possible explanation could be the so-called hitchhiking effect; this particular variant had a fitness advantage that was located outside the investigated region. For instance, resistance against any of the other antiretrovirals in the therapy regimen (e.g. efavirenz) could have been present in this variant but not in the other variants. While the chances that this occurs in one of the minority variants and not in any of the dominant species are small, the possibility cannot be excluded. Another possible explanation is that the variant with the N155H mutation is more fit than the E138K + Q148K mutant in the presence of raltegravir in this particular setting due to other factors (e.g. specific viral genetic background, immunological host factors, etc.).

We also observed a correlation between the number of unique sequence variants emerging and the magnitude of the virological response. Good virological response in Pts 1, 2 and 5 (> 2 log reductions in HIV-1 RNA) coincided with the number of unique sequence variants observed; the proportion of unique sequence variants increased when the viral load dropped. Pts 3 and 4 demonstrated moderate responses with viral load drops of around 1 log and showed no change in the relative frequencies of unique variants over the course of sampling. This suggests that in Pts 1, 2 and 5, treatment with raltegravir induced an elevated diversification to escape drug pressure while in Pts 3 and 4 the pressure on the population seemed to occur to a much lesser extent. This observation reminds of the explosive diversifications on a macro-evolutionary scale observed in other fields of biology after ice ages and the cataclysmic extinction of dinosaurs [47, 48]. However, regardless of treatment efficacy, i.e. both in patients with less dramatic and more severe virus load reductions, raltegravir resistance mutations developed and multiple resistance pathways were observed in 4/5 patients. The large extinction opens up previously occupied niches for new virus variants, (1) such that all new mutations are accepted and not compete for resources until the population regains a size limited by the carrying capacity of the system, or (2) most of the diversity pre-existed as a permanent, extremely low frequency pool of highly diverse viruses persisting in the shadow of more fit and high frequency variants. Once the high frequency variants are eradicated (e.g. by newly introduced drugs) a glimpse of that diversity surfaces. The larger the impact on the high frequency variants (i.e. reduction in viral load) the larger the proportion of the low frequency pool appears (i.e. number of unique variants increases when the viral load drops). Subsequently, one or two of the low frequency variants gain fitness in the new environment and become dominant, lowering the mean diversity again. The same can be argued from the point of viral escape. If the viral population doesn’t have to go that low to find fit variants (in the new environment), the impact on viral load is also less severe.

The raltegravir in vitro selections in which we mimicked population bottlenecking by using a low MOI allowing all resistant variants that are generated to emerge, clearly indicate that the resistance pathway that is selected to escape raltegravir pressure is not predisposed in the genetic background of the integrase CDS. Evaluation of the combined in vivo and in vitro data indicates that stochastic selection plays a major role during the initial development of raltegravir resistance.

In conclusion, the development and evolution of raltegravir resistance can be separated in multiple phases/components: (1) minority variants present at baseline can contribute to the emergence of raltegravir resistance but this is not preordained and seems to occur arbitrarily; (2) during the viral load drop due to drug pressure a burst of new sequence variants emerges creating diversifying selection; (3) these new variants usually include multiple raltegravir resistant variants (from multiple resistance pathways) that can pass the imposed bottleneck; (4) competition of these resistant variants determines the ultimate shape of the viral population. The resistant population can be the product of a single variant that outcompetes all others and only one variant represents the population or multiple variants with a similar fitness emerge that coexist in the viral population.

Further investigation is needed to better assess the exact impact of baseline minority resistance variants and the population size on the development and evolution of raltegravir resistance and determine the clinical implications of these factors.

## Conclusions

Emergence of resistance against integrase inhibitor raltegravir in HIV-1 patients is generally associated with selection of one of three distinct resistance pathways. The mechanisms that drive selection of a specific pathway are still poorly understood. Using deep sequencing we observed an inverse correlation between the virological response and the relative amount of unique sequence variants emerging, suggesting diversifying selection during drug pressure. In 4/5 patients multiple signature mutations representing different resistance pathways were observed. In addition, in vitro selections revealed that identical viral clones were also able to select mutations from different resistance pathways indicating that raltegravir resistance is not predisposed in the genetic background of the viral integrase. Importantly, raltegravir resistance develops progressively and discontinuation during early phases of therapy failure is justified to preserve future options with second-generation INSTIs.

## Methods

### Genotypic analysis

#### Population sequencing

HIV-1 RNA was isolated using the Nuclisens Islolation kit (BioMérieux, Boxtel, The Netherlands). Briefly, 100 μl of sample was mixed with 900 μl lysisbuffer and 40 μl silica and incubated for 10 min at room temperature to allow binding of the nucleic acid to the silica particles. Unbound material was removed by several washing steps after which the RNA was eluted at 56 °C with 100 μl of 40 ng/µl poly-A RNA. The isolated viral RNA was used to reverse transcribe and amplify the viral integrase coding region in a single-step reaction using the Titan One Tube RT-PCR kit (Roche). The RT-PCR was conducted with primers 5′INoutF1 (5′-GGA ATC ATT CAA GCA CAA CCA GA-3′; 4059–4081) and 3′INoutR1 (5′-TGT ATG CAG ACC CCA ATA TGT TG-3′; 5262–5241). The amount of amplified product was further enhanced in a second PCR using the Expand High fidelity kit (Roche) with primers 5′INinF1 (5′-TAT CTG GCA TGG GTA CCA GCA C-3′; 4143–4164) and 3′INinR1 (5′-TAG TGG GAT GTG TAC TTC TGA AC-3′; 5217–5195). All PCR-amplified products were purified using the QIAquick PCR purification kit (Qiagen, Leusden, The Netherlands). Sequence analysis was performed with the BigDye Terminator v3.1 Cycle Sequencing Kit (Applied Biosystems, Foster City, CA, USA). Integrase sequences were obtained using six primers: Intseq1 (5′-ATT GGA GGA AAT GAA CAA GT-3′; 4173–4192), Intseq2 (5′-AGC AGA AGT TAT TCC AGC AG-3′; 4484–4503), INT-3 (5′-TTC GGG TTT ATT ACA G-3′; 4897–4912), INT-4 (5′-CTT GTA TTA CTA CTG C-3′; 4986–4971), Intseq-5 (5′-CTG GCT ACA TGA ACT GCT AC-3′; 4470–4452) and 3′INinR2 (5′-GCT TTC ATA GTG ATG TCT ATA AAA CC-3′; 5178–5153). Sequence editing and contig assembly were performed using SeqScape v2.6 (Applied Biosystems) with HXB2 as a reference sequence.

#### Next-generation sequencing and data analysis

To examine the mutation frequencies within the viral integrase by pyrosequencing, RNA was reverse transcribed and amplified in a single step in a touch-down PCR using bar-coded primers to enable 454 pyrosequencing with pooled amplicons. The integrase core domain from amino acid position 53 to amino acid 180 was analyzed. All mutations in the viral integrase at 18 different codons associated with raltegravir resistance [16, 20, 23, 45, 49–52] were evaluated and included amino acids: T66, L68, V72, L74, E92, Q95, T97, F121, E138, G140, Y143, Q148, V151, N155, K156, E157, K160 and G163.

All amplicons were purified with AMPure magnetic beads (Agencourt, Beckman Coulter, Krefeld, Germany), quality checked and quantified using an Agilent 2100 Bioanalyser (Agilent Life Sciences, Waldbronn, Germany) and picogreen using the fluorometer Fluostar Optima (BMG Labtech, Offenburg, Germany), respectively. After equimolar pooling of the amplicons, emulsion PCRs were performed. Pyrosequencing was done using primers A and B (Titanium emPCR kit Lib-L v2; Roche-454 Life Sciences, Branford, CT, USA). After bead recovery and enrichment, approximately 250,000 beads per pool were loaded on one region of a GS FLX PicoTiter plate subdivided with a four-lane gasket. Pyrosequencing was performed on a Genome Sequencer FLX (Roche-454 Life Sciences). Sequence readings from the 454 pyrosequencing run were extracted directly from the Standard-Flowgram-Files (sff). Reads were pair-wise aligned against the integrase sequence of reference strain HXB2. Multiple mutations present in a single read were assumed to originate from the same genome.

The dynamics of the sequence populations were investigated on de-aligned sets to avoid artifacts due to inconsistent alignment gap placements using statistical functions in R [53]. For the analyses of the dominant variants in the populations, the redundancy-level for calling a variant was set at 80, i.e. only variants detected at least 80 times were considered. This level resulted in a strict filter that removed all known 454-sequencing artifacts [54–56]. The resulting dominant variants were aligned using MAFFT [57] and distance matrices were estimated using the R library ape [58] under a F84 substitution model (the choice of substitution model had no effect on the subsequent analyses). The R library sna [59] was used to construct minimum spanning trees (MSTs) based on the distance matrices of patient’s HIV population. Nodes were scaled according to both viral load and the relative abundance of each detected variant at time of sampling. We tracked known resistance mutations on the edges and edge lengths were drawn arbitrary in order to make the resulting graphs easy to look at.

### Construction of deletion clone HXB2∆INT

An HXB2 molecular clone (pHXB2AF) was used to construct a molecular deletion clone lacking the integrase coding region. pHXB2AF is derived from pHXB2WT [60], which expresses the full length HIV-1 sequence HXB2 (9719 bp, Genbank accession number K03455.1), with all bacterial sequences non-essential for bacterial expression and replication removed.

The NdeI site present in Gp120 (at HXB2 nt 6404) was inactivated to create a unique NdeI site at the 3′ end of the integrase CDS. Therefore pHXB2AF was digested with NcoI (Roche Diagnostics, Almere, The Netherlands) and NheI (New England Biolabs, Ipswich, MA, USA) to remove a 1586 bp fragment containing the NdeI site. PCR, using Vent_R_^®^ DNA polymerase (New England BioLabs) was performed on pHXB2AF with primers, NcoI-out (5′ CAC TAG AGC TTT TAG AGG AGC TTA AGA-3′; 5614–5640), NheI-out (5′-TTT TAT TAT TTC CAA ATT GTT CTC TTA-3′; 7296–7270) and NdeI-KO (5′-TCA GAT GCT AAA GCG TAT GAT ACA G-3′; 6390–6414). Primer NdeI-KO contained one silent nucleotide change (underlined) to inactivate the NdeI site in the PCR fragment. The amount of amplified product was further increased and enriched by performing a second amplification using Vent_R_^®^ DNA polymerase with primers NcoI-in (5′-GAG CTT TTA GAG GAG CTT AAG AAT GAA-3′; 5619–5645), NheI-in (5′-ATT GTT CTC TTA ATT TGC TAG CTA TCT-3′; 7281–7255) and NdeI-KO. This PCR fragment was then digested with NcoI and NheI and ligated with the digested pHXB2AF, resulting in pHXB2AFNdeIKO which was confirmed by sequence analysis of the complete fragment.

Subsequently, the integrase coding region was removed from pHXB2AFNdeIKO. Therefore pHXB2AFNdeIKO was digested with MluNI (Roche) and NdeI (New England BioLabs). The fragment between MluNI and the 5′ end of integrase was restored by performing a PCR on pHXB2AF using primers RT19 (5′-GGA CAT AAA GCT ATA GGT ACA G-3′; 2454–2472) and NgoMIV-INTlinker (5′-TAA TAT CAT ATG GAC AGC GTC GCC GGC ACT GAC TAA TTT ATC TAC TTG TTC-3′). Primer NgoMIV-INTlinker contained two silent nucleotide changes with respect to pHXB2AFNdeIKO, thereby introducing a unique NgoMIV site (underlined 4209–4214) in the PCR fragment. In addition to these nucleotide changes the primer NgoMIV-INTlinker contains a linker sequence with a unique NdeI site and an AspI site. AspI was used to prevent re-ligation of the vector and the linker. The amount of amplified product was further increased and enriched by performing a second amplification using Vent_R_^®^ DNA polymerase with primers RT19new2 (5′-GGA CCT ACA CCT GTC AAC ATA ATT GG-3′; 2484–2509) and NgoMIV-INTlinker. The PCR fragment was then digested with MluNI and NdeI and ligated with the digested pHXB2AFNdeIKO, resulting in a molecular deletion clone lacking the integrase coding region (pHXB2AFΔINT), which was confirmed by sequencing the complete fragment.

### Generation of recombinant virus

To generate recombinant viruses, the second PCR as described in the population sequencing section, was performed with a different primer pair: forward primer NgoMIV-Int2 (5′-TTA GTC AGT GCC GGC ATC AGG AAA G-3′; 4200–4224) which contains a NgoMIV restriction site (underlined) and reverse primer 3INinR2. The obtained integrase fragment and vector pHXB2AFΔINT were digested with restriction enzymes NgoMIV (New England BioLabs) and NdeI. The PCR product and vector pHXB2AFΔINT were ligated overnight at 4 °C using the Rapid Ligation System (Promega, Benelux, Leiden, The Netherlands). After ligation, plasmids were digested with AspI and subsequently transformed into competent cells. Bacteria were cultured overnight at 37 °C. Plasmid isolation was performed using the QIAgen Plasmid Mini kit (Qiagen).

Viruses were generated by transfecting 293T cells with 10 ug plasmid DNA using Lipofectamine 2000 reagent (Invitrogen) according the manufacturer’s protocol. Two days after infection cell free virus was harvested and the viral infectivity (TCID_50_) was determined using end-point dilutions in MT2 cells.

### Viral and cell culture

#### Cells

SupT1 and MT-2 cells were maintained in RPMI 1640 medium with l-glutamine (Lonza, Verviers, Belgium) supplemented with 10% FBS (FBS; Sigma-Aldrich, Zwijndrecht, The Netherlands) and 10 µg/ml gentamicin (Invitrogen, Breda, The Netherlands). 293T cells were maintained in DMEM with l-glutamine (Lonza) supplemented with 10% FBS and 10 µg/ml gentamicin.

#### In vitro selection experiments

The raltegravir in vitro selection experiments with two recombinant viruses that contained patient-derived integrase CDS and HIV-1 reference strain HXB2 were each performed 5 times. The in vitro selections were initiated by infecting 2 × 10^6^ SupT1 cells at a multiplicity of infection (MOI) of 0.001. The initial raltegravir concentration was 2 nM Raltegravir. Cultures were monitored daily for cytopatic effect (CPE) and twice a week half of the medium was replaced by fresh medium supplemented with raltegravir. When full blown CPE was observed, cell free virus was harvested. The raltegravir concentration was raised in each passage to a final concentration of 1024 nM in passage 10. After passage 10, HIV-1 RNA was isolated from all cultures for genotypic analysis. As a control, in vitro evolution experiments (10 passages) were performed with HXB2 reference strain (5 individual cultures) to monitor the evolution of the integrase CDS during culture in absence of inhibitor.

#### Phenotypic drug susceptibility analysis

Drug susceptibility was determined by a multiple cycle cell-killing assay [61]. MT-2 cells (4 × 10^4^ in 200 µl RPMI 10% FBS per well) were plated in 96-well microplates. Sample virus and reference virus were inoculated for five days on a single 96-well plate in the presence of threefold dilutions of raltegravir. Both sample virus and reference virus were inoculated at multiple MOIs to adjust for any differences in viral RC. Fold change (FC) values were calculated by dividing the mean 50% inhibitory concentration (EC_50_) for a sample virus by that of the HXB2 reference strain.
